# Phase I / II trial of dose adjusted EPOCH chemotherapy with bortezomib combined with integrase inhibitor therapy for HTLV-1 associated T-cell leukemia lymphoma

**DOI:** 10.1186/1742-4690-12-S1-P21

**Published:** 2015-08-28

**Authors:** Lee Ratner, JC Ramos, Ariela Noy, Stefan Barta, Samir Parikh, Adrienne Phillips, Richard Ambinder, Dan Rauch, John Harding, Hicham Baydoun, Xiaogang Cheng, Breanna Caruso, Steven Jacobson

**Affiliations:** 1 Division of Oncology, Washington University, St Louis, MO, USA; 2 University of Miami, Miami, FL, USA; 3 Lymphoma & Hematology Division, Memorial Sloan Kettering Cancer Center, New York, NY, USA; 4 Divisions of Hematology-Oncology, Montefiore Hospital, New York, NY, USA; 5 Columbia University, New York, NY, USA; 6 Division of Hematologic Malignancies, Johns Hopkins University, Baltimore, MD, USA; 7 NINDS, NIH, Bethesda, MD, USA

## 

Adult T-cell leukemia lymphoma (ATLL) acute and lymphoma subtypes have a poor prognosis, with median survival of about one year. The malignant cells are characterized by high levels of nuclear factor kappa B (NFkappaB). In order to improve therapy, we assessed the safety, tolerance, and efficacy of a combination of dose-adjusted EPOCH chemotherapy (etoposide, prednisone, vincristine, cyclophosphamide, and doxorubicin), combined with proteasome inhibitor, bortezomib, to prevent degradation of the inhibitor of NFkappaB (IkappaB). In addition, integrase inhibitor, raltegravir, was added to the regimen to block virus replication occurring during treatment. This multicenter study enrolled 18 of 20 planned subjects over 2.5 yrs in the U.S., although 15 of the subjects were born in the Caribbean. Six subjects had acute ATLL, and the remainder lymphoma subtype, all but one with stage 4 disease. Therapy was well tolerated; subjects received 1-6 cycles of therapy (mean 4.5 cycles). Two subjects achieved complete remission lasting for >12 mos, 10 subjects had a partial remission, and 3 subjects had stable disease as their best response. Baseline calcium level, absolute lymphocyte count, and proviral DNA load were not predictive of response. Correlations with proviral expression, integration site, and integrase gene analyses will be presented. Supported by NIH grants CA94056, CA1730, CA63413, Lymphoma Leukemia Society grant 6067-10, and Lymphoma Research Foundation grant 307181203.

